# Promoting cell proliferation and collagen production with ascorbic acid 2-phosphate-releasing poly(l-lactide-co-ε-caprolactone) membranes for treating pelvic organ prolapse

**DOI:** 10.1093/rb/rbae060

**Published:** 2024-05-24

**Authors:** Alma Kurki, Kaarlo Paakinaho, Markus Hannula, Sanna Karjalainen, Kirsi Kuismanen, Jari Hyttinen, Susanna Miettinen, Reetta Sartoneva

**Affiliations:** Biomedical Technology (TECH) Research Unit, Faculty of Medicine and Health Technology (MET), Tampere University, 33520 Tampere, Finland; Tays Research Services, Tampere University Hospital, Wellbeing Services County of Pirkanmaa, 33520 Tampere, Finland; Biomedical Technology (TECH) Research Unit, Faculty of Medicine and Health Technology (MET), Tampere University, 33520 Tampere, Finland; Tays Research Services, Tampere University Hospital, Wellbeing Services County of Pirkanmaa, 33520 Tampere, Finland; Biomedical Technology (TECH) Research Unit, Faculty of Medicine and Health Technology (MET), Tampere University, 33520 Tampere, Finland; Biomedical Technology (TECH) Research Unit, Faculty of Medicine and Health Technology (MET), Tampere University, 33520 Tampere, Finland; Department of Obstetrics and Gynaecology, Tampere University Hospital, 33520 Tampere, Finland; Biomedical Technology (TECH) Research Unit, Faculty of Medicine and Health Technology (MET), Tampere University, 33520 Tampere, Finland; Biomedical Technology (TECH) Research Unit, Faculty of Medicine and Health Technology (MET), Tampere University, 33520 Tampere, Finland; Tays Research Services, Tampere University Hospital, Wellbeing Services County of Pirkanmaa, 33520 Tampere, Finland; Biomedical Technology (TECH) Research Unit, Faculty of Medicine and Health Technology (MET), Tampere University, 33520 Tampere, Finland; Tays Research Services, Tampere University Hospital, Wellbeing Services County of Pirkanmaa, 33520 Tampere, Finland; Department of Obstetrics and Gynaecology, Wellbeing Services County of South Ostrobothnia, 60220 Seinäjoki, Finland

**Keywords:** ascorbic acid 2-phosphate, poly(l-lactide-co-ε-caprolactone), tissue engineering, adipose-derived stem/stromal cell, pelvic organ prolapse

## Abstract

Pelvic organ prolapse (POP) afflicts millions of women globally. In POP, the weakened support of the pelvic floor results in the descent of pelvic organs into the vagina, causing a feeling of bulging, problems in urination, defaecation and/or sexual function. However, the existing surgical repair methods for relapsed POP remain insufficient, highlighting the urgent need for more effective alternatives. Collagen is an essential component in pelvic floor tissues, providing structural support, and its production is controlled by ascorbic acid. Therefore, we investigated novel ascorbic acid 2-phosphate (A2P)-releasing poly(l-lactide-co-ε-caprolactone) (PLCL_A2P_) membranes *in vitro* to promote cell proliferation and extracellular matrix protein production to strengthen the natural support of the pelvic fascia for POP applications. We analysed the mechanical properties and the impact of PLCL_A2P_ on cellular responses through cell culture analysis using human vaginal fibroblasts (hVFs) and human adipose-derived stem/stromal cells (hASCs) compared to PLCL. In addition, the A2P release from PLCL_A2P_ membranes was assessed *in vitro*. The PLCL_A2P_ demonstrated slightly lower tensile strength (2.2 ± 0.4 MPa) compared to PLCL (3.7 ± 0.6 MPa) for the first 4 weeks *in vitro*. The A2P was most rapidly released during the first 48 h of *in vitro* incubation. Our findings demonstrated significantly increased proliferation and collagen production of both hVFs and hASCs on A2P-releasing PLCL_A2P_ compared to PLCL. In addition, extracellular collagen Type I fibres were detected in hVFs, suggesting enhanced collagen maturation on PLCL_A2P_. Moreover, increased extracellular matrix protein expression was detected on PLCL_A2P_ in both hVFs and hASCs compared to plain PLCL. In conclusion, these findings highlight the potential of PLCL_A2P_ as a promising candidate for promoting tissue regeneration in applications aimed for POP tissue engineering applications.

## Graphical abstract

**Figure:**
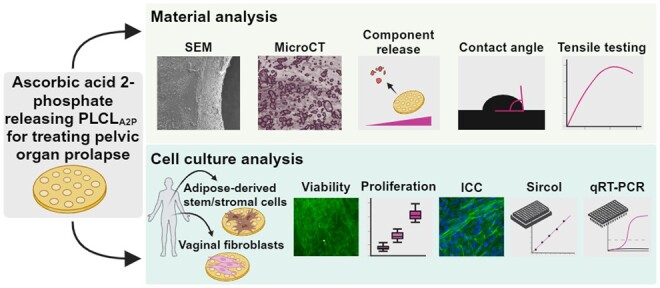

## Introduction

Pelvic organ prolapse (POP) is a highly common problem affecting millions of women worldwide, and some form of prolapse is detected among 40% of women aged 50–79 years during gynaecological examination [1, 2]. Further, the lifetime risk for a woman to require a surgery due to POP is even up to 13% [3] and over 300 000 POP operations are performed annually in the USA alone [4, 5]. POP can be divided to a prolapse of an anterior and/or posterior vaginal wall and/or uterus or vaginal vault. Primarily, POP are operated using patient’s own fascia. However, the probability of recurrence is high, up to 30% [4, 6]. Until recently, the recurrent POP has been reconstructed with nonabsorbable polypropylene (PP) meshes. However, the incidence of overall complications, such as infections, pelvic pain, sexual problems, vaginal shrinkage, mesh exposure and migration, is high, even up to 15–25% [7, 8]. Therefore, the US Food and Drug Administration first issued two warnings, followed by classifying vaginal PP meshes as high-risk products and ordered market withdrawal of anterior meshes in 2019 [8, 9]. As a result of these reasons, as well as negative publicity surrounding vaginal meshes, most manufacturers have withdrawn vaginal PP meshes from the market, leading to a high demand for new surgical treatment methods and biomaterials for POP surgery. Therefore, tissue engineering and biodegradable biomaterials have emerged as appealing options for future POP surgery.

Tissue engineering combines biodegradable biomaterials, cells and active molecules to restore the damaged tissue by also recruiting the intrinsic tissue healing mechanism. The biomaterial for POP should be biocompatible, elastic, flexible, suturable and have mechanical properties close to the natural pelvic fascia to bear the load caused by the abdominal pressure while the *de novo* fascia retains its strength. For POP applications, two-dimensional (2D) membrane or mesh designs are feasible alternatives to complex three-dimensional (3D) structures due to their easier manufacturing, handling and ability to tailor mechanical properties to meet load-bearing requirements. The biomaterial should integrate into the surrounding tissue and promote the growth and collagen production of stromal cells to restore the support of the pelvic fascia. Additionally, the material should be able to be manufactured on a large scale with uniform quality and standardized mechanical properties. The poly(l-lactide-co-ε-caprolactone) (PLCL) is an aliphatic polyester meeting all the above-mentioned criteria, making it highly appealing material, especially for soft tissue engineering applications. Previous studies have shown its potential in vascular, oesophageal, urethral and vaginal tissue engineering [10–17]. Electrospun PLCL/fibrinogen mesh for anterior POP surgery has been used in two clinical pilots with potential results [18, 19]. However, according to our knowledge, those are the only previously published studies utilizing PLCL for POP tissue engineering.

In addition to scaffold design, cell-seeding is considered to further accelerate tissue integration time and reduce the inflammatory response following mesh implantation [20, 21]. Fibroblasts are the primary cell type of the pelvic floor and therefore previous POP tissue engineering research has mainly utilized dermal or oral fibroblasts but also muscle-derived progenitor cells and endometrial progenitor cells [22–26]. For gynaecological applications, human vaginal fibroblasts (hVFs) are an application-specific cell type, and in addition to vaginal tissue engineering, they have also been studied for POP applications [15, 27, 28]. In addition to primary cells, adipose-derived stem/stromal cells (ASCs) are an attractive cell type due to their ability to differentiate into multiple cell types, including smooth muscle cells, to promote local progenitor cells and angiogenesis, and reduce excessive inflammatory reactions [29, 30]. These unique characteristics, in addition to their ease of isolation, have sparked interest in studying ASCs as a cell source for POP applications, with promising preliminary results [21, 31, 32].

The weakening of the connective tissue fascia and the alterations in the collagen Type I (COL I) and III (COL III) ratio are considerable factors behind POP. The main challenge in POP tissue engineering is to achieve an adequate and permanent increase in the strength of *de novo* fascia after biomaterial degradation. One fundamental solution would be to promote the synthesis of collagen in the surrounding tissue by embedding bioactive components and/or cell-seeding in the tissue-engineered scaffolds. Previously, ascorbic acid (AA) has not only been demonstrated to play an essential role in collagen synthesis but also to increase cell proliferation, maturation and stem cell differentiation [33–35]. The disadvantage of AA is its instability and therefore more stable derivatives of AA, such as ascorbic acid 2-phosphate (A2P), have emerged as a potential option for scaffold incorporation, yet the number of published studies is relatively low. Previously, A2P-embedded 3D supercritical carbon dioxide-foamed porous scaffolds have been studied for urothelial and vaginal tissue engineering to promote human adipose-derived stem/stromal cell (hASC) proliferation and collagen production [12, 36]. Furthermore, only one previous study has been published on the potential of A2P-embedded electrospun PLA scaffolds based on their ability to promote dermal fibroblast proliferation and collagen production *in vitro* for POP applications [24].

In this study, we studied A2P-releasing PLCL membranes as a novel alternative for POP applications. Our aim was to investigate the effect of A2P-releasing PLCL membranes on hVF and hASC viability, proliferation, collagen and other extracellular matrix (ECM) protein production, and to assess the membrane tensile strength to support the native tissue in clinical application. Moreover, we aimed to investigate the efficacy of hASCs as an alternative cell type to hVFs for POP applications.

## Materials and methods

### Membrane manufacturing

The studied membranes were manufactured by melt-mixing the A2P (Sigma-Aldrich) and PLCL (70 L/30 CL, Corbion, Gorinchem, the Netherlands) in a twin-screw extrusion process with the mixing ratio of 7% of A2P and 93% of the polymer. The plain PLCL were produced without the addition of A2P. Next, the rods were cut and compression moulded into a 0.35 ± 0.05 mm sheet, which were further punched to a dog bone shape according to ISO 3167 with adapted values (sample test length 25 mm and width 5 mm) or laser cut into 12-mm diameter discs for cell culture with (hPLCL, hPLCL_A2P_) or without (PLCL, PLCL_A2P_) 1.2-mm diameter perforations. The two sides of the membranes were similar and there were no distinguishable top and bottom surfaces. The samples were ethanol washed, vacuum dried and gamma irradiated with the minimum dose of 25 kGy.

### Scanning electron microscopy

The PLCL, hPLCL, PLCL_A2P_ and hPLCL_A2P_ membranes used in the cell culture experiments were imaged with scanning electron microscopy (SEM) to assess the membrane surface microstructures. The samples were sputtered with 5 nm of platinum/palladium and imaged at a voltage of 5 kV using Zeiss ULTRAPlus SEM microscope (Zeiss, Oberkochen, Germany).

### Component release

The release of A2P from the PLCL matrix in an *in vitro* environment was analysed with UV-VIS-NIR spectrophotometer (UV-3600 Plus, Shimadzu). The calibration curves were performed by dissolving known amounts of A2P to Sörensen buffer solution (SBS) prepared according to ISO 13781:2017. The A2P release was assessed from 12 mm discs submerged in 10 ml SBS placed in an incubator shaker (100 rpm) at 37°C for a period of 4 weeks (*n* = 6). Buffer solution was analysed and changed at time points (3 h, 6 h, 24 h, 48 h, 72 h, 1 week, 2 weeks, 3 weeks and 4 weeks).

### Micro-computed tomography imaging

The micro-computed tomography (µCT) images were acquired using MicroXCT-400 (Xradia, Zeiss, Pleasanton, CA, USA) device. A total of 1601 projections were captured with 2 s exposure time. The source voltage was set at 60 kV, and the source current was 167 µA. The 3D model was reconstructed using Zeiss XMReconstructor software, resulting in a pixel size of 5.64 µm. A2P particle analysis was conducted for three Day 0 samples. Initially, particles were segmented through manual thresholding, followed by the measurement of volumes for individual particles. Particle analysis and 3D visualizations were performed using Avizo 3D 2023.1.1 software (Thermo Fisher Scientific, Waltham, MA, USA).

### Water contact angle measurements

The hydrophobicity of the PLCL_A2P_ and PLCL membranes was determined with contact angle measurements. The membranes were randomly oriented for the measurements, as the top and bottom sides for the membranes are non-distinguishable. Contact angles (*n* = 64) were measured with sessile drop technique (drop size 3.4 µl) using Attention Theta Lite optical tensiometer (Biolin Scientific, Espoo, Finland).

### Tensile testing

SBS pH 7.4 was prepared following the ISO 13781:2017. The gamma-irradiated PLCL_A2P_ and PLCL dog bones (*n* = 6) were submerged in 10 ml SBS and incubated in +37°C for 1 day, 2 weeks, 4 weeks, 6 weeks, 8 weeks, 10 weeks or 12 weeks. The buffer solution was changed weekly. At time points, the samples were tensile tested submerged in +37°C water bath (Instron CP106108) using Instron Electropuls E1000 (Instron, Norwood, MA, USA) with a 250 N load cell. The loading rate was 30 mm min^−1^ and gauge length 12 mm.

Further, to determine the mass loss during the *in vitro* hydrolysis, the samples were dry-weighed prior to submerging in SBS (*n* = 4–6). After the tensile test, sample pieces were carefully collected and dried for 1 week in a fume hood followed by 1 week of drying in a vacuum. After drying, samples were dry-weighed again to determine the mass loss during hydrolysis. The mass loss percentage was calculated by subtracting the mass of the dried test sample (*W_d_*) from the initial dry mass of the test sample (*W_0_*) and then dividing by the initial mass of the test sample:

(1) $$\text{Mass loss percentage }=\;\left(W_{0}-W_{d}\right)/W_{0}\times \text{1}00\%$$

### Ethical considerations

hVFs and human adipose stromal cells used in this study were obtained from three different donors during elective surgery from Tampere University Hospital, with the supportive statements of the Ethics Committee of Pirkanmaa Hospital District (R15051 and R15161, respectively) and donors’ written consent.

### Cell isolation

The hVFs were isolated as described previously by Sartoneva *et al*. [15]. Briefly, the tissue was cut into small pieces and incubated in a solution containing 1.5 mg/ml collagenase Type I (Life Technologies, Thermo Fisher Scientific, Waltham, MA, USA) and 4 mg/ml dispase (Invitrogen, Thermo Fisher Scientific) in a water bath. The suspension was filtered, centrifuged and plated to CellBind T75 flask (Sigma-Aldrich, St Louis, MO, USA) with EpiLife medium (Invitrogen) supplemented with 1% EpiLife defined growth supplement (EDGS; Invitrogen), 0.1% of CaCl_2_ (Invitrogen) and 0.35% of antibiotics (100 U/ml penicillin and 0.1 mg/ml streptomycin; Lonza, BioWhittaker, Verviers, Belgium). After primary culturing, the hVFs were separated from the epithelial cell line by treating the cells with TrypLE Select (Gibco, Thermo Fisher Scientific) and the loosened cells were passaged to T75 flasks (Nunc, Thermo Fisher Scientific) in DMEM/F12 (Thermo Fisher Scientific) supplemented with 5% human serum (HS; Biowest, Nuaillé, France), 1% GlutaMAX (Life Technologies) and 1% of antibiotics (100 U/ml penicillin and 0.1 mg/ml streptomycin; Lonza), and cultured in a humidified atmosphere with 5% of CO_2_. The hVFs were passaged when confluent and cryopreserved until experiments. The hVFs in Passages 3 or 4 were used in the *in vitro* experiments.

Isolation of hASCs was done as previously described [37]. Briefly, the adipose tissue sample was manually cut to smaller pieces, digested with collagenase NB 6 (SERVA Electrophoresis GmbH, Heidelberg, Germany), centrifuged and filtered to separate the hASCs from the surrounding tissue. Thereafter, the hASCs were cultured in αMEM medium (Lonza) supplemented with 5% HS (Biowest) and 1% penicillin/streptomycin (Lonza) in T75 cell culture flasks (Thermo Fisher) in a humidified atmosphere with 5% CO_2._ The isolated hASCs were passaged when confluent and subsequently cryopreserved until the experiment. Expression of cell surface markers of the used donor hASC lines are presented in Table 1. Overall, hASCs in Passage 3 from three donors were used.

**Table 1.** Cell surface marker expression of hASCs used in the current study^a^

		Cell surface marker expression (%)							
**Donor line**	**P**	**CD14**	**CD19**	**CD34**	**CD45**	**CD73**	**CD90**	**CD105**	**HLA-DR**
ASC 1	2	7.1	7.7	11.5	8.7	99.5	99.8	100	1
ASC 2	2	3.2	3.7	19.5	4.8	98	100	100	0.7
ASC 3	2	0.9	0.8	51.3	3.4	93.3	99.4	99.8	1.2

a Analysis was done for cells in passage (P) 2 to confirm the presence of stem/stromal cells and to ensure the hASC isolation protocol

### Cell-seeding

Membranes PLCL, hPLCL, PLCL_A2P_ and hPLCL_A2P_ were placed to 24-well plate in a random orientation and fixed with Cellcrown^TM^ inserts (Scaffdex Oy, Tampere, Finland). The polystyrene (PS) well bottom served as a control material in the cell culture experiments. The perforated hPLCL and hPLCL_A2P_ membranes represent a preliminary design to be used in living tissues for efficient tissue integration. The nonperforated PLCL and PLCL_A2P_ membranes were included in the cell experiments to maximize the available surface area for cell attachment for better data collection on the materials’ effects on cell responses, without considering the influence of the membrane design. The membranes were prewetted in 1 ml of αMEM cell culture medium for 24 h prior to cell-seeding. Thereafter, the medium was changed, and the cells were seeded on membranes in 30 µl medium. Cell concentrations of 5000 cells/well for quantitative assays and 7000 cells/well for qualitative assays were used for hVFs. The hASCs were seeded in 2000 cells/well for all assays.

### Cell viability

The viability of hVFs and hASCs were evaluated with qualitative live/dead fluorescence staining at Days 7 and 14 time points (*n* = 4) as described previously [13]. Briefly, the cells were incubated in a mixture of 0.25 mM calcein AM (Thermo Fisher) and 0.3 mM ethidium homodimer-1 (Thermo Fisher) for 45 min at RT. Thereafter, the cells were imaged with a fluorescent microscope (IX51, Olympus, Tokyo, Japan). Viable cells were stained green, and dead cells were stained red. Materials without cells were used to exclude the false-positive staining caused by the materials.

### Cell proliferation

The CyQUANT Cell Proliferation Assay kit (Thermo Fisher) was used to assess the proliferation of hVFs and hASCs after 1, 7 and 14 days of cell culture by determining the relative amount of DNA on the samples. Briefly, 0.1% Triton-X lysis buffer (Sigma-Aldrich) was used to lyse the cells and the lysate was stored in –70°C until analysis. After thawing, 20 µl of sample and 180 µl of a working solution containing CyQUANT GR dye and lysis buffer were pipetted to two parallel wells of 96-well plate. The fluorescence was measured at 480/520 nm with a microplate reader (Victor 1420 Multilabel Counter; Wallac, Turku, Finland). The analysis was performed for the cells from three different donors with three parallel samples for each material, and three technical replicates (*n* = 18–27). Relative cell numbers were calculated relative to Day 1 mean result of one donor cell line.

### Total collagen content

After 14 days of culture, the amount of total soluble collagen content of hVFs and hASCs on PLCL, hPLCL, PLCL_A2P_, hPLCL_A2P_ and control PS was evaluated using Sircol Soluble Collagen Assay (Biocolor, Carrickfergus, UK) measuring mammalian Type I–V collagen as described previously [38]. Briefly, to extract the acid-soluble collagen, the samples were incubated in 0.5 M acetic acid (Merck, Darmstadt, Germany) with 0.1 mg/ml pepsin (Sigma-Aldrich) for 4 h at +4°C. Thereafter, Sircol Dye reagent (Biocolor) was added and the samples were incubated with gentle shaking for 30 min at RT. This was followed by centrifugation for 10 min at 12 000 rpm and washing the collagen pellets with ice-cold Acid-Salt Wash Reagent (Biocolor). The centrifugation was repeated, and the dyed collagen was further dissolved in 0.5 M sodium hydroxide solution (Biocolor). Two parallel samples were pipetted to the 96-well plate, and the red dye intensity at 540 nm was measured with Victor 1420 microplate reader (Wallac). The cells from three different donors, three parallel samples and two technical replicates (*n* = 16–18) for each material were used in the analysis.

### Quantitative real-time polymerase chain reaction

Quantitative real-time reverse transcription-polymerase chain reaction (qRT-PCR) was used to evaluate the expression of genes COL I, COL III, elastin and α-SMA on Day 14 of culture. Analysis was performed as previously described [39]. Following cell lysis and total RNA isolation using Nucleospin kit reagent (Macherey-Nagel GmbH & Co. KG, Düren, Germany), the mRNA was reverse transcripted to cDNA with high-capacity cDNA Reverse Transcriptase Kit (Thermo Fisher Scientific). The qRT-PCR mixture contained cDNA, forward and reverse primers (Table 2), and SYBR green PCR Master Mix (Applied Biosystems, CA, USA). The AbiPrism 7000 Sequence Detection System (Applied Biosystems) was used to conduct the reaction. The initial enzyme activation was performed at 95°C for 10 min, followed by 45 cycles of denaturation at 95°C for 15 s and annealing and extension at 60°C for 60 s. The results were normalized to the expression of housekeeping gene large ribosomal protein P0 (RPLP0) and the relative expression was calculated using previously described mathematical model [40]. Three parallel samples were pooled together for each donor cell line to obtain adequate amount of RNA for the analysis. Results were calculated from technical duplicates or triplicates of each sample (*n* = 6–9). The mRNA quantities determined with mean Ct values were used in the calculation of COL I/III ratio.

**Table 2.** Primers used in the qRT-PCR analysis of hVFs and hASCs cultured on PLCL, hPLCL, PLCL_A2P_ and hPLCL_A2P_ membranes

Name	Primer	Sequence 5'–3'	Product size (bp)	Accession number
*RPLP0*	F	AAT CTC CAG GGG CAC CATT	70	NM_001002
	R	CGC TGG CTC CCA CTT TGT		
*α-SMA*	F	GAC AAT GGC TCT GGG CTC TGT AA	194	NM_001613.4
	R	ATG CCA TGT TCT ATC GGG TAC TT		
*elastin*	F	GGT GCG GTG GTT CCT CAG CCT GG	613	NM_000501.4
	R	GGG CCT TGA GAT ACC CCA GTG		
*COL I*	F	CCA GAA GAA CTG GTA CAT CAG CAA	94	NM_000088.3
	R	CGC CAT ACT CGA ACT GGA ATC		
*COL III*	F	CAG CGG TTC TCC AGG CAA GG	179	NM_000090
	R	CTC CAG TGA TCC CAG CAA TCCC		

### Immunofluorescent staining

The hVF and hASC expression of α-SMA (ab7818, anti-actin α-smooth muscle, 1:200, Abcam) and COL I (ab90395 anti-collagen I antibody, 1:2000, Abcam, Cambridge, UK) was evaluated after 14 days on PLCL, hPLCL, PLCL_A2P_, hPLCL_A2P_ or PS control (*n* = 4). Briefly, the samples were fixed with 0.2% Triton X-100 (Sigma Aldrich) in 4% PFA (Paraformaldehyde; Sigma Aldrich) and incubated overnight in the above-mentioned primary antibody dilutions at +4°C. The following day, the cells were incubated at RT in secondary antibody dilutions (green fluorescence; A11029 1:300 or A21121 1:400, respectively). Finally, the cell nuclei were stained with DAPI (blue fluorescence; 1:2000, Invitrogen), and the samples were imaged with a fluorescence microscope (Olympus). The false-positive staining and the non-specific staining were excluded using non-seeded membranes and cell-seeded membranes without primary antibody, respectively.

### Statistical analysis

Statistical analysis was performed using IBM SPSS Statistics version 29 (IBM Corp., Armonk, NY, USA). Kruskal–Wallis test or Mann–Whitney *U* test for non-normally distributed data was used to evaluate the statistical significance of the results. Values of *P* < 0.05 were considered significant. Unadjusted *P*-values are presented in the Results Section. If preferred, Bonferroni post-hoc adjusted *P*-values are obtained by multiplying the unadjusted *P*-values by 10.

## Results

### Membranes in cell experiments

**Figure 1.**
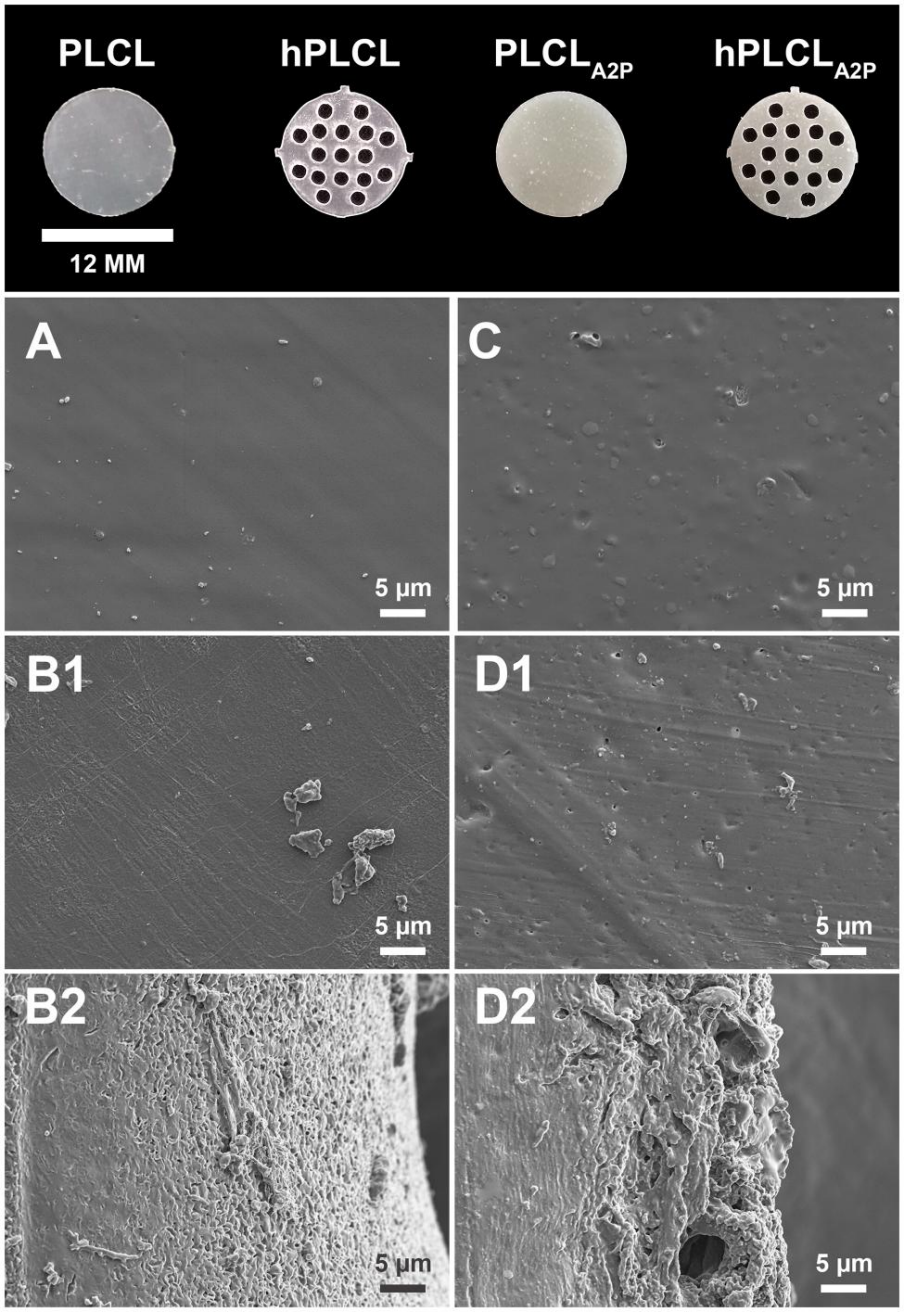
Top row shows photographs of the 12-mm diameter membranes used in the cell culture experiments. In the SEM image panel (**A**) PLCL, (**B1**) hPLCL flat area next to perforations, (**B2**) hPLCL laser-cut perforation edge, (**C**) PLCL_A2P_, (**D1**) hPLCL_A2P_ flat area next to perforations and (**D2**) hPLCL_A2P_ laser-cut perforation edge. Scale bar 5 µm.

Photographs and SEM images of the PLCL, hPLCL, PLCL_A2P_ and hPLCL_A2P_ membranes used in the cell experiments are presented in Figure 1. The microarchitecture on all membranes appeared reasonably similar, with slightly rougher surface on PLCL_A2P_ and hPLCL_A2P_ membranes compared to PLCL and hPLCL, due to the embedded A2P particles in the polymer matrix. Some additional surface irregularities are visible on both hPLCL and hPLCL_A2P_ resulting from the laser cutting for creating the perforated design.

### A2P release peaked in the initial phase and decreased PLCL hydrophobicity

**Figure 2.**
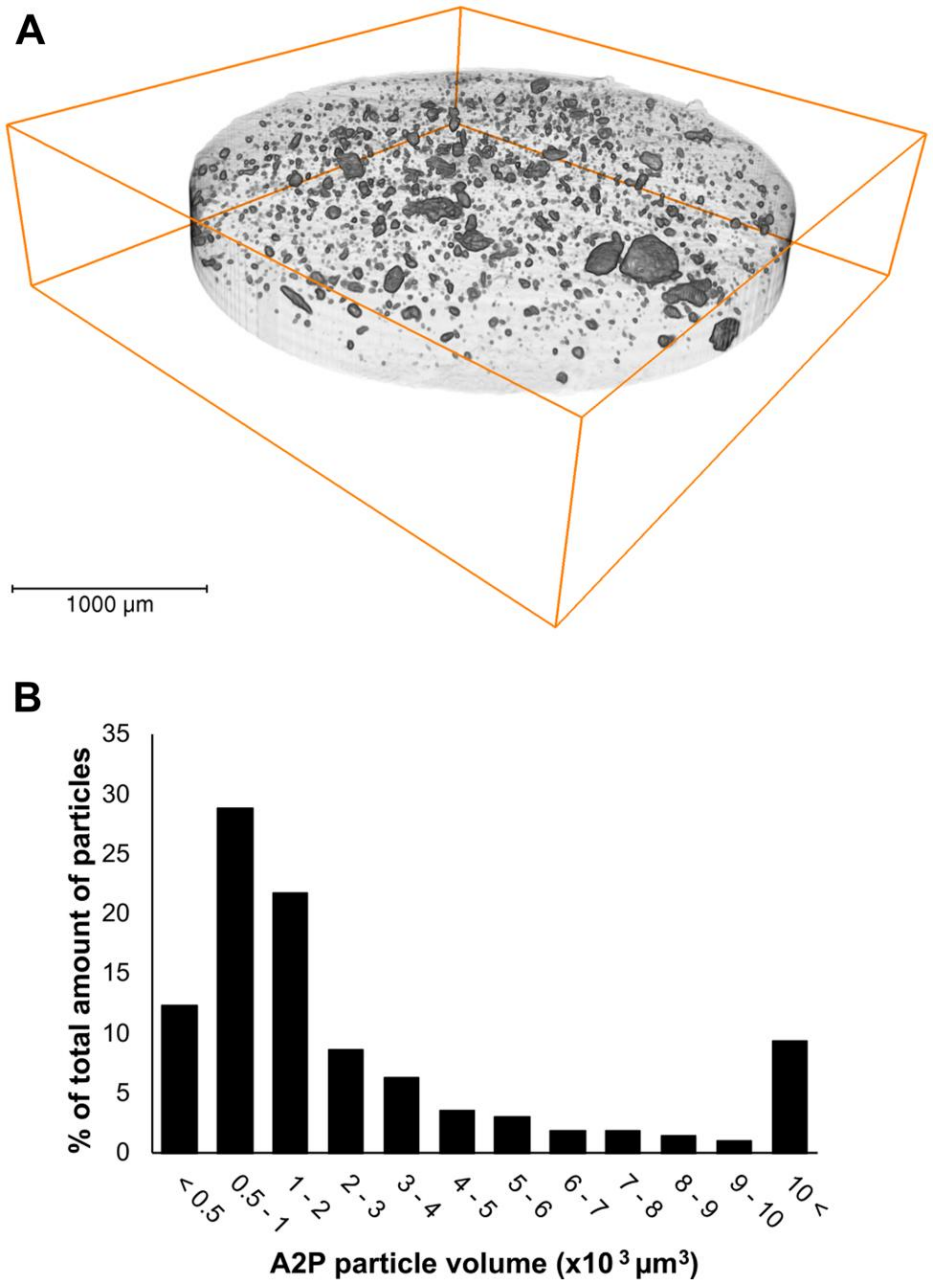
(**A**) Example of A2P particle distribution within PLCL_A2P_ membrane. (**B**) Distribution of A2P particle volumes (*n* = 3).

Particle distribution in PLCL_A2P_ membranes were determined with µCT imaging (Figure 2). The minimum and maximum A2P particle volumes were 360 µm^3^ and 98 600 µm^3^, respectively, and 50% of embedded A2P were between 500 and 2000 µm^3^.

**Figure 3.**
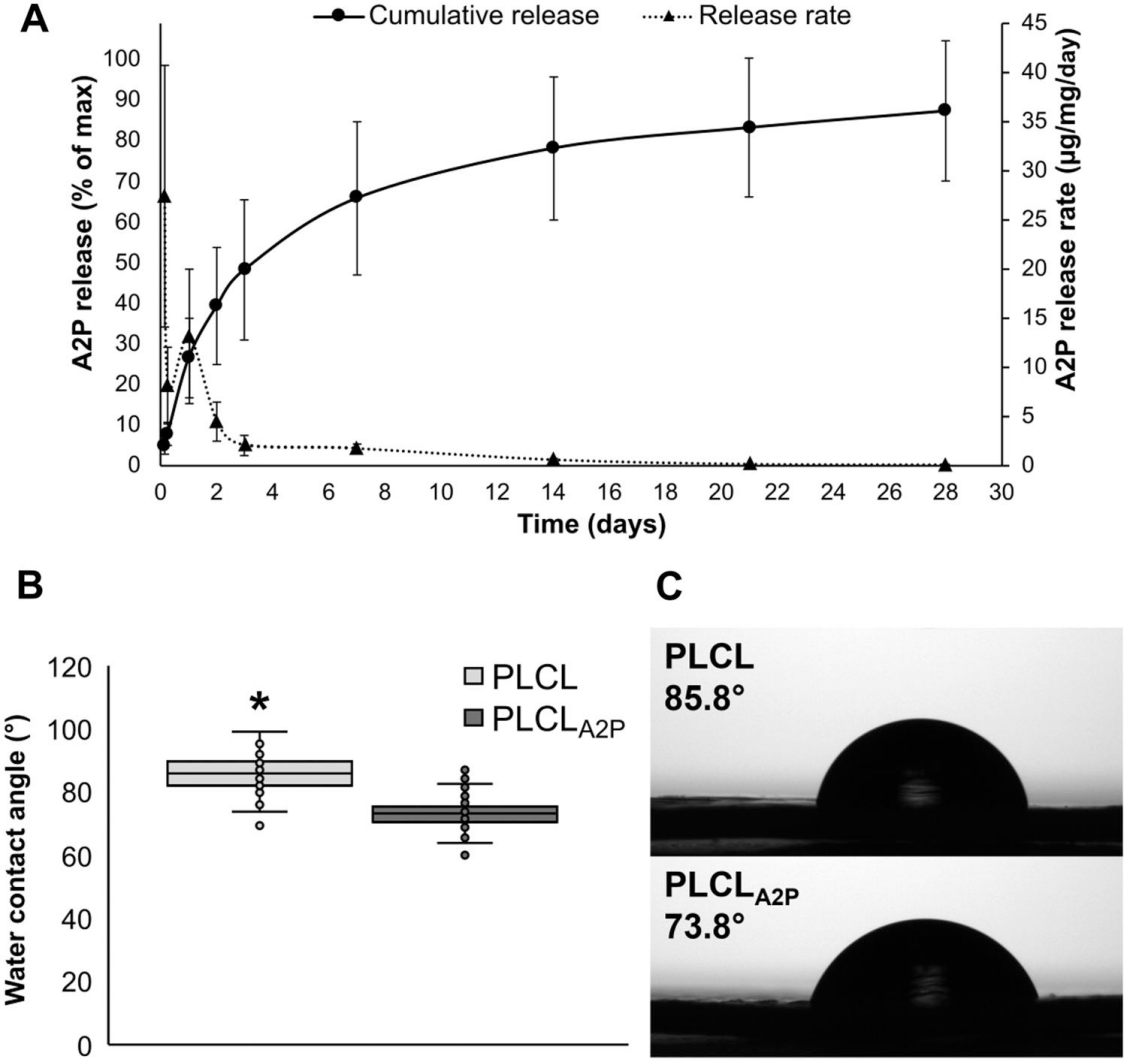
The A2P release and the effect to hydrophobicity. (**A**) Cumulative A2P release (% of max) and A2P release rate (µg/mg/day) from PLCL_A2P_ membranes. Error bars represent the standard deviation. *n* = 6. (**B**) The average contact angles were significantly higher on PLCL than on PLCL_A2P,_ statistical significance tested with Mann–Whitney *U* test. *P <0.001, *n* = 64. (**C**) Example images with mean contact angle values.

Figure 3A presents the release rate and cumulative release of A2P from the PLCL_A2P_*in vitro* at 37°C. The cumulative release of A2P during the 4-week *in vitro* period was 87 ± 26% of the initial A2P content mixed with the PLCL polymer. The A2P release rate fluctuated during the first 2 days having the fastest calculated release rate of 27.4 ± 13.3 µg/mg/day during the first 3 h. The release rate declined steadily after the first 3-day period reaching the daily release rate of 0.1 ± 0.02 µg/mg/day at the end of the 4-week study period.

Embedded A2P decreased PLCL hydrophobicity. Mean ± SD contact angles were 85.8 ± 5.6° (min 69.5°, max 99.4°) for plain PLCL and 73.8 ± 5.5° (min 60.3°, max 87.6°) for PLCL_A2P_ (*P* < 0.001) (Figure 3B and C).

### A2P affected initial tensile properties

**Figure 4.**
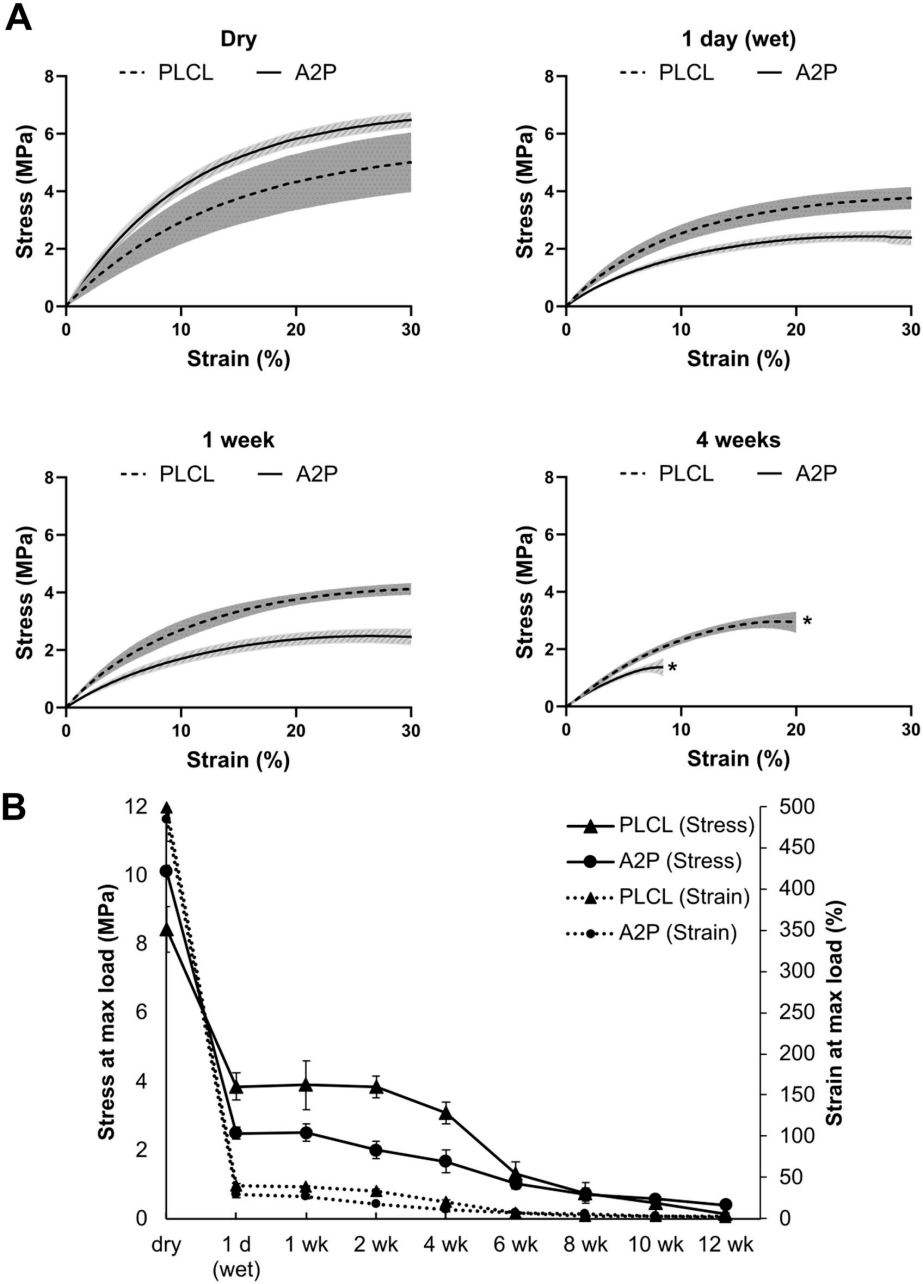
(**A**) Average ± SD stress–strain curves of PLCL and PLCL_A2P_ (A2P) up to a 30% strain. * = at Week 4, curves are plotted until first parallel sample is fractured. *n* = 6. (**B**) PLCL and PLCL_A2P_ (A2P) dog bone average stress and strain at maximum load during the tensile test series. Error bars represent the standard deviation. *n* = 5–6 (*n* = 3–4 on Weeks 8–12 due to the sample degradation).

PLCL and PLCL_A2P_ stress–strain curves at selected time points (dry, 1 day (wet), Week 1, Week 4) until 30% strain are presented in Figure 4A. The stress curves of both PLCL and PLCL_A2P_ are considerably decreased after commencing *in vitro* hydrolysis, as seen between the dry samples and Day 1 wet (after 24 h in SBS) samples. Dry PLCL_A2P_ has higher stress curve compared to PLCL but decreases below PLCL after wetting on Day 1. The stress–strain curves for both materials remain reasonably stable between Day 1 and Week 1. At 30% strain, the stress values for Day 1 wet samples are 3.8 ± 0.4 MPa for PLCL and 2.4 ± 0.3 MPa for PLCL_A2P_. At Week 4, the strength of the materials has significantly decreased, and the first parallel sample for PLCL is fractured at 20% strain (2.7 MPa) and at 8% strain (1 MPa) for PLCL_A2P_.

Figure 4B depicts the stress and strain at maximum load of PLCL and PLCL_A2P_ during the 12-week *in vitro* tensile test series. The effect of wetting is evident, as the stress at max load decreased from 8.4 ± 0.7 MPa of a dry sample to 3.9 ± 0.4 MPa of 1 day (wet) sample in PLCL and from 10 ± 0.2 MPa to 2.5 ± 0.2 MPa for PLCL_A2P_. The stress curves progress similarly over time, but for the first 4 weeks, the stress values of PLCL_A2P_ are ∼1.5 MPa less than those of PLCL. Similarly, the strain at maximum load decreases from 498 ± 1% of a dry PLCL sample to 40 ± 2% on Day 1, and from 480 ± 26% for dry PLCL_A2P_ sample to 29 ± 4% on Day 1. For 4 weeks, the strain remains higher for PLCL by ∼11%. The stress and strain at maximum load of PLCL decrease to the level of the PLCL_A2P_ after 6 weeks *in vitro*, being 1–1.3 MPa and 7% at 6-week time point.

The dog bones were dry-weighed prior to incubation and post-tensile testing after vacuum drying. At the 1-week time point, the mass loss was significantly higher (*P* = 0.002) for PLCL_A2P_ (10 ± 0.2%) than for PLCL (0.8 ± 0.4%). Mass loss at 4- and 8-week time points was still higher (*P* = 0.002) for PLCL_A2P_ than PLCL, 11.2 ± 0.7% and 2.2 ± 0.3%, 17.6 ± 3.3% and 8 ± 0.9%, respectively. The last reliable time point for mass loss was Week 10, at which point, the mass loss of PLCL and PLCL_A2P_ was similar (*P* = 0.914), 26.3 ± 7.2% and 27.9 ± 5.5%, respectively.

### Both hVFs and hASCs remain viable on PLCL_A2P_ and hPLCL_A2P_ membranes

**Figure 5.**
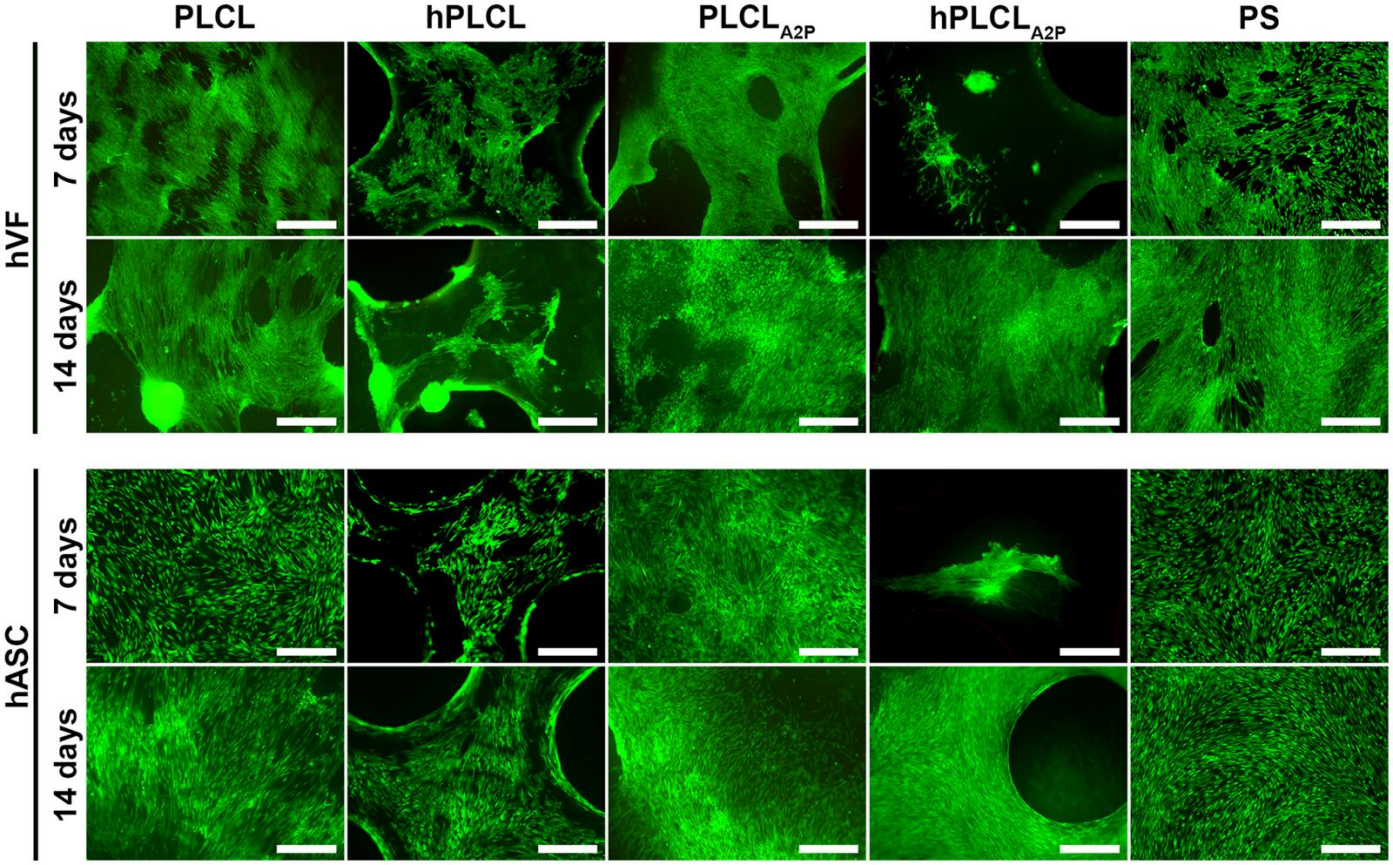
Viability of hVFs and hASCs on PLCL, hPLCL, PLCL_A2P_, hPLCL_A2P_ or control PS was assessed with live/dead assay on Days 7 and 14 time points. Both hVFs and hASCs remained viable on all materials and no dead cells were detected. Scale bar 500 µm.

The viability of hVFs and hASCs cultured on PLCL, hPLCL, PLCL_A2P_, hPLCL_A2P_ or control PS was evaluated at Days 7 and 14 time points (Figure 5). Both cell types remained viable on all materials and a negligible number of dead cells were detected. Cell clustering was seen in hVFs on PLCL and hPLCL membranes, but not on other materials. No clustering was seen in hASCs on any material.

### Cell proliferation increased on PLCL_A2P_ and hPLCL_A2P_

**Figure 6.**
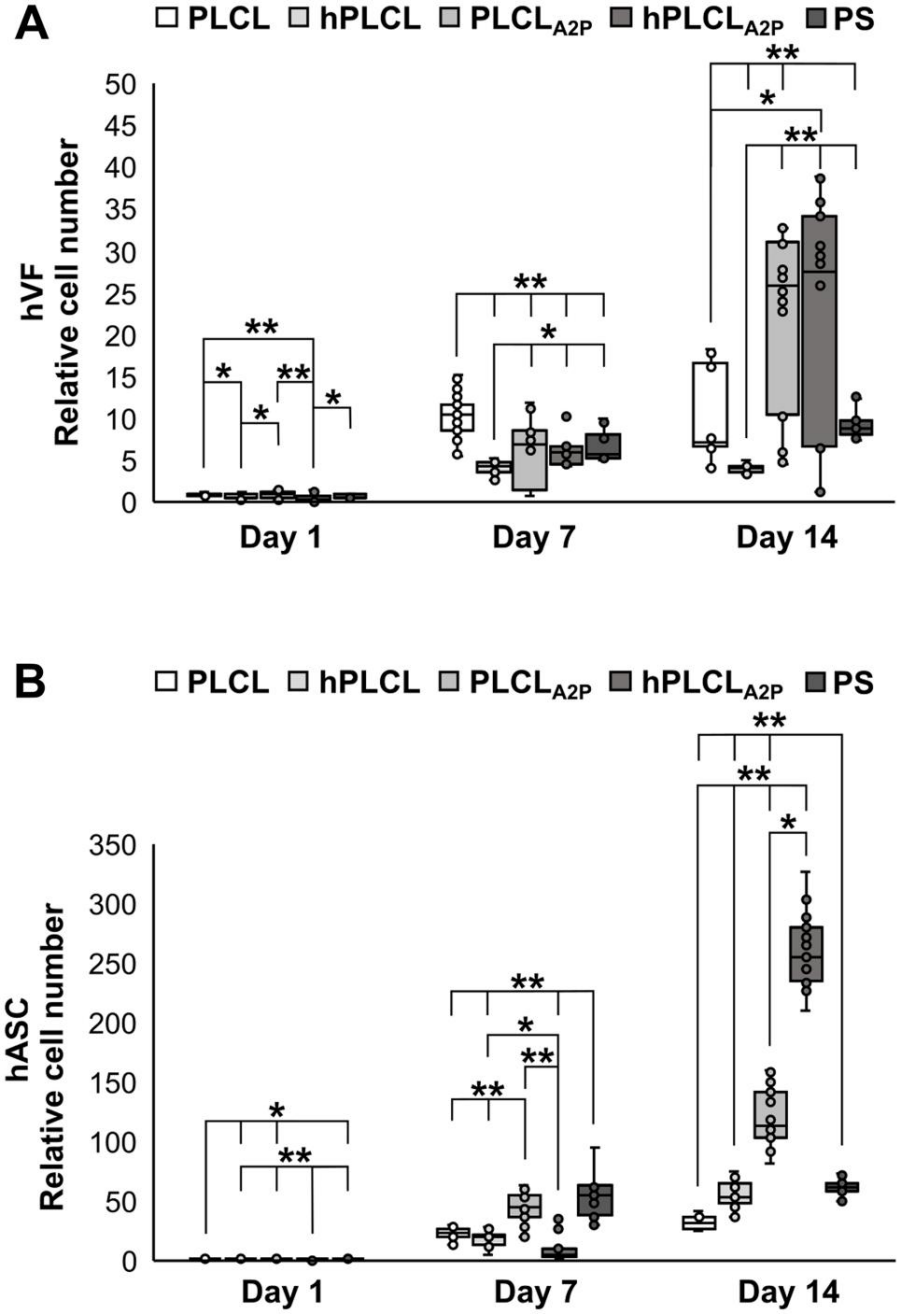
Relative cell number on Day 14 was increased on A2P-releasing PLCL_A2P_ and hPLCL_A2P_ membranes in (**A**) hVF cultures and (**B**) hASC cultures. Statistical significance tested with Kruskal–Wallis test. *P < 0.05, **P <0.001, *n* = 18–27.

Cell proliferation of hVFs and hASCs on PLCL, hPLCL, PLCL_A2P_, hPLCL_A2P_ and PS control was measured after 1, 7, and 14 days of cell culture. The highest proliferation was observed on the PLCL_A2P_ and hPLCL_A2P_ membranes for both cell types (Figure 6A and B)_._ Number of hVFs continuously increased on PLCL_A2P_, hPLCL_A2P_ and PS control during the 14-day assessing period (*P* < 0.001), whereas no significant hVF increase was detected between Days 7 and 14 on PLCL or hPLCL. On Day 1 the hVF amount was low, yet the highest relative number of hVFs was on PLCL_A2P_, with a significant difference to hPLCL (*P* = 0.025), hPLCL_A2P_ (*P* < 0.001) and PS (*P* = 0.043). More hVFs were measured on PLCL compared to hPLCL (*P* = 0.014) and hPLCL_A2P_ (*P* < 0.001). On Day 7, the relative number of hVFs was the highest on PLCL (*P* < 0.001). Moreover, the lowest hVF proliferation was detected on hPLCL compared to the rest of the materials; PLCL_A2P_ (*P* = 0.005), hPLCL_A2P_ (*P* = 0.027) and PS (*P* = 0.003). On Day 14, hVF proliferation was significantly higher on the A2P-releasing PLCL_A2P_ and hPLCL_A2P_ membranes compared to PLCL (*P* < 0.001 and *P* = 0.03, respectively) and hPLCL (*P* < 0.001). Like on Day 7, the hVF number on Day 14 was the lowest on hPLCL (*P* < 0.001).

The hASCs proliferated on all materials, and the cell numbers significantly increased during the 14-day cell culture period (*P* < 0.001). However, on PS, the hASC increase was not significant between days 7 and 14. Number of hASCs on Day 1 was similar between hPLCL, PLCL_A2P_ and PS control with no significant differences. The hASC number was higher on hPLCL, PLCL_A2P_ and PS compared to PLCL (*P* = 0.019, *P* = 0.005 and *P* = 0.01, respectively) and to hPLCL_A2P_ (*P* < 0.001). On Day 7, the highest hASC number was detected on PS control (*P* < 0.001); however, the difference was not significant between PS and PLCL_A2P_ (*P* = 0.214). However, the hASC proliferation on Day 7 was higher on PLCL_A2P_ than on PLCL, hPLCL or hPLCL_A2P_ (*P* < 0.001). Notably, the hASC proliferation on Day 14 was superior on hPLCL_A2P_ (*P* = 0.011 compared to PLCL_A2P_ and *P* < 0.001 compared to the other materials), while the second highest hASC proliferation was detected on PLCL_A2P_ (*P* < 0.001). The hASC number on Day 14 was the lowest on PLCL (*P* < 0.001). No other significance could be detected.

### hVFs produced extracellular COL I on PLCL_A2P_ and hPLCL_A2P_

**Figure 7.**
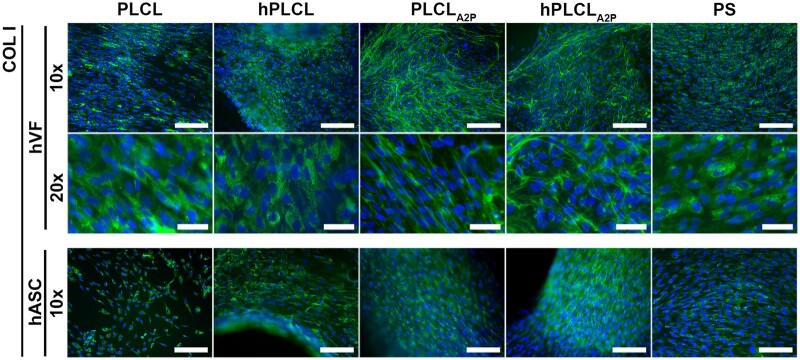
Immunofluorescent images of COL I staining in hVFs and hASCs on Day 14. Extracellular COL I fibres could be seen in hVFs on PLCL_A2P_ and hPLCL_A2P_. Higher magnifications (20×) of hVF COL I staining show the COL I in the extracellular space more clearly. No extracellular COL I could be seen in hASCs. Scale bars are 200 µm for 10× and 50 µm for 20×.

Immunofluorescent staining of α-SMA and COL I was performed on Day 14. Staining of COL I was seen in hVFs on all materials (Figure 7). Strikingly, extracellular fibrous COL I was detected in hVFs on PLCL_A2P_ and hPLCL_A2P_ membranes. No such fibres were seen in hVFs on other materials, where COL I appeared to be intracellular. In hASCs, COL I is stained on all materials, yet no extracellular COL I fibres can be seen.

**Figure 8.**
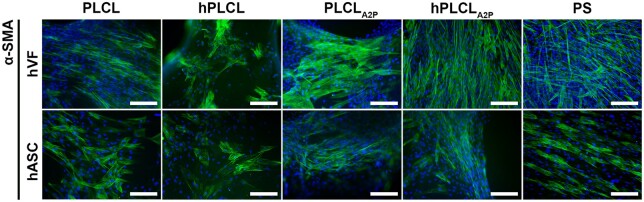
Immunofluorescent images of α-SMA staining in hVFs and hASCs. The hVFs on PLCL_A2P_, hPLCL_A2P_ and PS control appear to stain more α-SMA. In hASCs, the α-SMA staining appears similar on all materials. Scale bar 200 µm.

The α-SMA is observed in hVFs and hASCs on all materials (Figure 8). Especially the PLCL_A2P_ and hPLCL_A2P_ appear to support α-SMA in hVFs. In hASCs, α-SMA staining between the studied materials appears similar and no definite conclusions can be drawn.

### Collagen production was enhanced in hASCs on PLCL_A2P_ and hPLCL_A2P_

**Figure 9.**
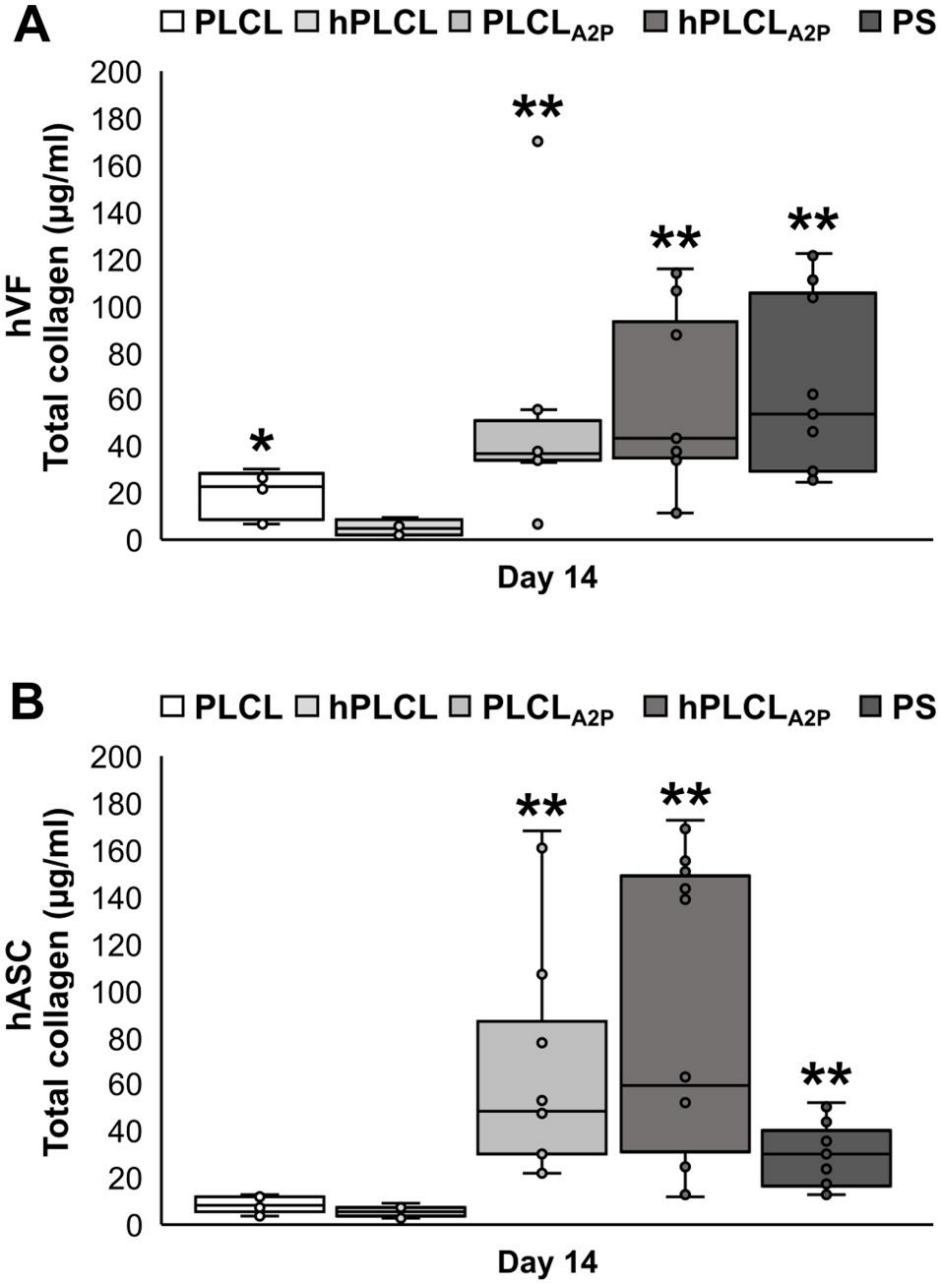
Total amount of collagen (µg/ml) on Day 14 in (**A**) hVF cultures and (**B**) hASC cultures. Statistical significance tested with Kruskal–Wallis test. * = higher to hPLCL (*P* = 0.05), ** = higher to PLCL and hPLCL (*P* < 0.01). *n* = 16–18.

The total amount of soluble collagen (Types I–V) produced in hVF and hASC cultures on PLCL, hPLCL, PLCL_A2P_, hPLCL_A2P_ or control PS was measured with quantitative Sircol analysis on Day 14. Collagen production of both hVFs and hASCs was increased on PLCL_A2P_ and hPLCL_A2P_. For hVFs (Figure 9A), the amount of collagen was reasonably similar between PLCL_A2P_, hPLCL_A2P_ and PS, yet significantly higher compared to PLCL (*P* = 0.003, *P* < 0.001 and *P* < 0.001, respectively) and hPLCL (*P* < 0.001). In addition, the amount of total collagen was higher on PLCL compared to hPLCL (*P* = 0.041). The mean ± SD collagen amounts for hVF cultures were 18.7 ± 9.5 µg/ml on PLCL, 5.4 ± 2.9 µg/ml on hPLCL, 51.8 ± 46.6 µg/ml on PLCL_A2P,_ 57.2 ± 34.1 µg/ml on hPLCL_A2P_ and 64.9 ± 35.6 µg/ml on PS. Although the highest amount of hVF total collagen was measured on the PS control, there was no statistically significant difference between the PS controls and PLCL_A2P_ and hPLCL_A2P_ membranes.

Total collagen produced by hASCs was significantly higher on PLCL_A2P_, hPLCL_A2P_ and control PS compared to PLCL and hPLCL (*P* < 0.001) (Figure 9B). No other statistical significances could be detected. The mean ± SD collagen amounts for hASC cultures were 8.3 ± 3.1 µg/ml on PLCL, 5.5 ± 1.9 µg/ml on hPLCL, 63.1 ± 44.2 µg/ml on PLCL_A2P,_ 83.8 ± 57.4 µg/ml on hPLCL_A2P_ and 29.7 ± 13.0 µg/ml on PS.

### Increased gene expression on hPLCL_A2P_ and PLCL_A2P_

**Figure 10.**
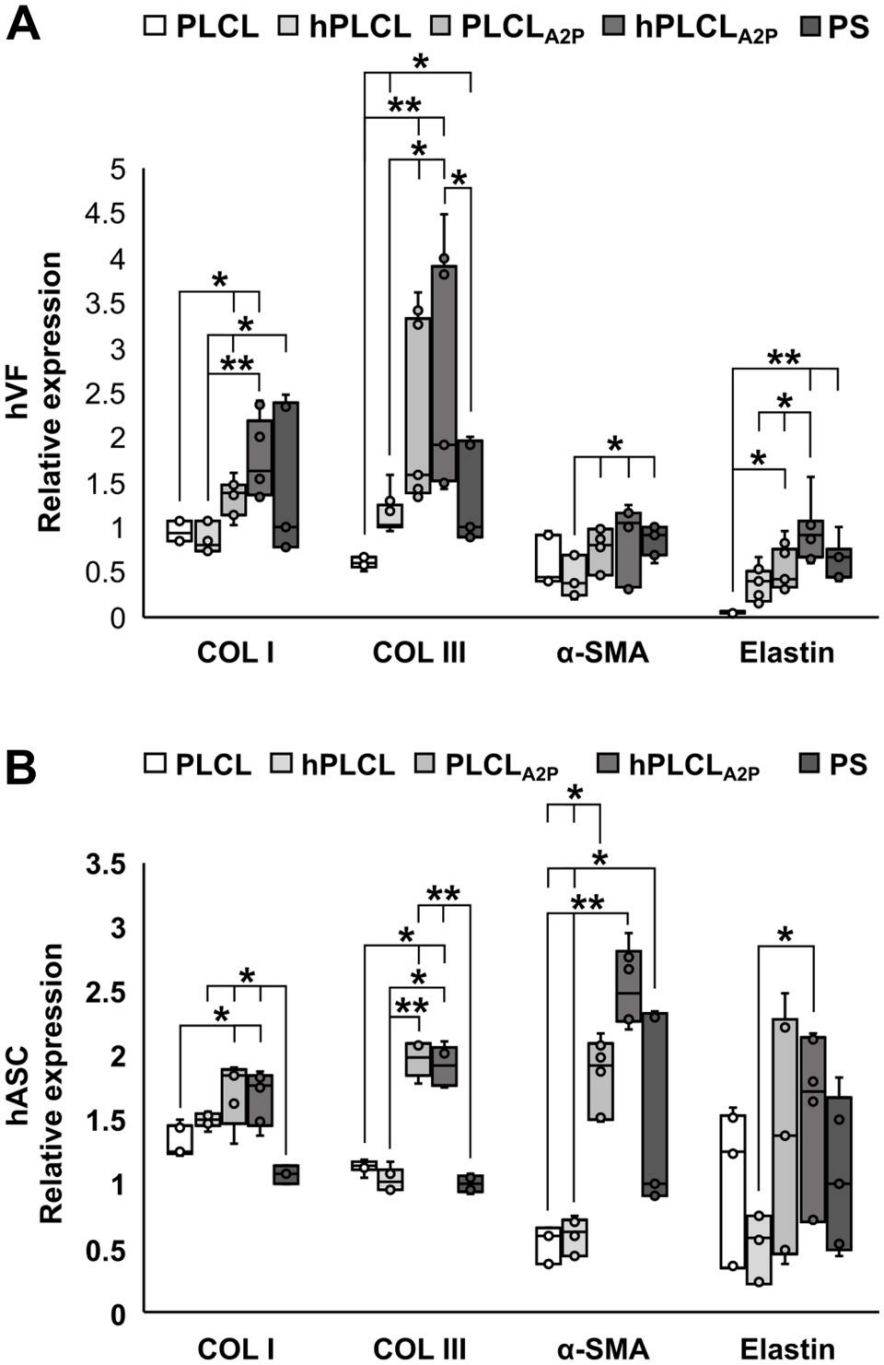
Relative expression of COL I, COL III, α-SMA and elastin in (**A**) hVF and (**B**) hASC cultures on Day 14. Statistical significance tested with Kruskal–Wallis test. * = *P* < 0.05, ** = *P* < 0.001, *n* = 6–9.

Cell expression of COL I, COL III, α-SMA and elastin on Day 14 was determined using qRT-PCR. For hVFs (Figure 10A), the amount of COL I mRNA was significantly higher on PLCL_A2P_ and hPLCL_A2P_ compared to PLCL (*P* = 0.05 and *P* = 0.003, respectively) and hPLCL (*P* = 0.004 and *P* < 0.001, respectively). Additionally, COL I expression was higher on PS compared to hPLCL (*P* = 0.048). Expression of COL III was also increased on PLCL_A2P_ and hPLCL_A2P_ membranes compared to PLCL (*P* < 0.001) and hPLCL (*P* = 0.029 and *P* = 0.006, respectively). In addition, the mean ratio of COL I/III mRNA was <1 on all studied membranes (0.73 on PLCL, 0.79 on hPLCL, 0.68 on PLCL_A2P_ and 0.74 hPLCL_A2P_), whereas on PS control, the ratio was 1.01. The amount of α-SMA mRNA was increased on PLCL_A2P_, hPLCL_A2P_ and PS compared to hPLCL (*P* = 0.016, *P* = 0.002, and *P* = 0.005, respectively). Elastin expression was significantly higher on hPLCL_A2P_ compared to other membranes; PLCL (*P* < 0.001), hPLCL (*P* = 0.002) and PLCL_A2P_ (*P* = 0.033). In addition, elastin expression was higher on PLCL_A2P,_ and PS compared to PLCL (*P* = 0.005 and *P* < 0.001, respectively). No other statistical significances were detected.

In hASCs (Figure 10B), the COL I expression was higher on PLCL_A2P_ and hPLCL_A2P_ compared to PLCL (*P* = 0.008 and *P* = 0.019, respectively) and to PS control (*P* = 0.001 and *P* = 0.003, respectively). In addition, COL I was higher on PLCL compared to PS (*P* = 0.023). Moreover, the hASC expression of COL III was superior on PLCL_A2P_ and hPLCL_A2P_ membranes compared to PLCL (*P* = 0.021 and *P* = 0.049, respectively), hPLCL (*P* < 0.001 and *P* = 0.003, respectively) and PS control (*P* < 0.001). The ratio of COL I/III mRNA was the lowest on PLCL_A2P_ and hPLCL_A2P_ membranes (0.58 and 0.56, respectively) and the highest on hPLCL (0.98). The ratio was 0.77 on PLCL and 0.70 on PS. Expression of hASC α-SMA was significantly higher on PLCL_A2P_ and hPLCL_A2P_ compared to PLCL (*P* = 0.009 and *P* < 0.001, respectively) and hPLCL (*P* = 0.023 and *P* < 0.001, respectively). In addition, α-SMA expression was higher on PS control compared to PLCL and hPLCL (*P* = 0.014 and *P* = 0.032, respectively). Statistical increase in elastin expression was detected on hPLCL_A2P_ compared to hPLCL (*P* = 0.012). No other statistical significances were detected.

## Discussion

We studied A2P-releasing PLCL_A2P_ and hPLCL_A2P_ membranes for POP applications and assessed the membrane tensile properties and their effect on hVF and hASC growth and ECM protein expression. Our findings suggest that PLCL_A2P_ exhibits suitable tensile properties for potential use in POP applications. Furthermore, our results reveal increased proliferation and collagen production of hVFs and hASCs on the novel A2P-releasing PLCL_A2P_ and hPLCL_A2P_ membranes. However, as the first *in vitro* study of the PLCL_A2P_ membranes, further research is required to design the implant used in clinical setting.

The exact strength of vaginal tissue and pelvic floor fascia are still unknown. In particular, the current knowledge on the mechanical properties of the weakened pelvic floor fascia in POP is insufficient. Yet, tensile strengths of 0.42–0.79 MPa [41, 42] and maximum elongation 17% [41] have been reported for non-prolapsed vaginal tissue. While we observed slightly decreased tensile properties for PLCL_A2P_ compared to PLCL in the first 4 weeks, both PLCL and PLCL_A2P_ can be considered to have appropriate tensile properties for POP applications [41, 42]. In contrast to our observations, Mangir *et al*. have previously reported A2P to increase the mechanical properties of wet electrospun PLA scaffolds [24]. We measured stress values over 1 MPa for 6 weeks for both PLCL and PLCL_A2P_ membranes. At Weeks 8 and 10, the stress values were declined to ∼0.7 and 0.5 MPa, respectively, which can still be considered sufficient. However, since the mechanical properties depend on the shape and design of the implant, more thorough material analysis is required when considering the final design of the potential device, which could resemble the conventionally used transvaginal PP meshes [43]. Mechanical properties closer to native tissue might alleviate the development of long-term complications, which can be partly attributed to the higher mechanical properties of the conventional PP meshes [44]. Nevertheless, the challenge for all biodegradable materials is their evident gradual loss of mechanical properties [45]. Therefore *in vivo*, seeded cells and embedded bioactive components might promote the tissue growth on site to compensate for the support lost by the degraded scaffold. Previously, Nair *et al*. demonstrated that the ECM proteins produced by dermal fibroblasts compensated for the loss of tensile strength following scaffold degradation [46].

To enhance tissue adhesion and remodelling, scaffolds coated or embedded with various components, such as growth factors, oestradiol and platelet-rich plasma, have been studied for POP applications [24, 28, 32, 47]. In this study, we investigated A2P, as AA is known to have an essential role in collagen synthesis [34]. However, previous research on polymer-embedded AA or its derivatives for soft tissue applications is highly limited [12, 24, 36]. Previously, the effect of AA and A2P on cell function has been mainly researched using medium supplementation [25, 48–50], which differs significantly from scaffold incorporation, where the embedded component is released continuously over time. In the current study, we specifically assessed the quantity of A2P released from PLCL_A2P_ membranes over a 4-week *in vitro* period. More rapid A2P release was observed for the first 72 h, during which 50% of the total A2P content was released. Afterwards, the release rate slowed down such that 87 ± 26% A2P was released during the 4-week period. Interestingly, compared to the Asikainen *et al.*, the release followed a similar trend despite the structural differences between the studied PLCL_A2P_ membranes and the 3D porous PLCL scaffolds of the previous study [51]. It has been previously shown that the presented release rates and subsequent A2P cellular lever concentrations are well in the level of cytocompatibility and biocompatibility [12, 36]. While the release of A2P peaked in the first days of incubation, the lasting impact on the cell function and ECM production persisted throughout the entire 2-week cell culture period.

As the release studies were conducted in a physiological buffer solution without cells, the A2P concentrations cannot be directly compared to a cell culture setting due to the continuous breakdown of A2P and consumption by the cells. The most reliable comparison between the release study and cell culture conditions can be done at Day 1 time point, when the measured concentration of the released A2P in buffer was 50 ± 20 µg/ml (160 ± 70 µM). Previously, supplemented 250 µM A2P was utilized to significantly enhance hASC proliferation to construct hASC sheets for tissue engineering applications [49], and 100 µg/ml supplemented AA resulted in the most competent ECM production in hASCs [52]. Moreover, 250 µM A2P increased proliferation of bone marrow-derived mesenchymal stromal cells [33]. It is important to note that excess AA and A2P have shown cytotoxic effects *in vitro* [33, 52, 53]. Concentrations over 200 µg/ml AA inhibited hASC proliferation and ceased completely in over 500 µg/ml AA [52]. However, higher cell density was found to protect hASCs from cytotoxic effects [53]. The current study is the first to use A2P-embedded PLCL membranes. The 7% A2P concentration for the PLCL_A2P_ and hPLCL_A2P_ membranes was selected based on our previous *in vitro* studies using 3D supercritical carbon dioxide-foamed PLCL scaffolds [12, 36, 51]. Despite the structural differences to the scaffolds in previous publications, the concentration proved to be suitable also for PLCL_A2P_ and hPLCL_A2P_ membranes. We did not detect any cytotoxic effects in our cell culture experiments, yet further optimization of A2P concentration may reveal further advantages in cell responses. The continuous release of A2P from the scaffold aims to maintain a steady A2P concentration to prevent cytotoxic concentrations and to maintain a steady effect of A2P on cells *in vitro,* and particularly *in vivo* and in clinical applications.

Excellent viability of hVFs and hASCs on PLCL_A2P_ and hPLCL_A2P_ membranes suggests good cytocompatibility, as expected based on the previous results [12, 14–16, 36]. Clustered hVFs can be observed on PLCL and hPLCL membranes but not on the A2P-embedded PLCL_A2P_ and hPLCL_A2P_. Similar hVF clustering was detected in our previous research on 3D porous supercritical carbon dioxide-foamed PLCL scaffolds [15, 36]. However, scaffold manufacturing process, surface structure, and area of the previously studied 3D porous PLCL scaffolds differ from the PLCL_A2P_ membranes of the current study. These differences may lead to different cell responses. The hydrophobicity of the PLCL surface could lead to cell clustering, which may interfere with adequate cell adhesion. We found that A2P significantly decreased the hydrophobicity of PLCL, which could enable better cell attachment to PLCL_A2P_ and hPLCL_A2P_. Embedded A2P has been reported to decrease hydrophobicity also in electrospun PLA polymer scaffolds [24]. Previously, improved cell attachment to hydrophilic surfaces has been reported for hASCs [54]. However, we did not observe hASC clustering on any of the studied membranes, contrary to the results of our previous study, where hASCs formed clusters on supercritical carbon dioxide-foamed 3D PLCL scaffolds yet spread evenly on A2P-embedded 3D porous PLCL scaffolds [12]. These differences may be attributed to the structural differences between the 2D PLCL membranes and 3D porous PLCL scaffolds.

In potential clinical application, an implanted membrane is aimed to integrate with the native tissue and provide enhanced support. By promoting cell proliferation, PLCL_A2P_ and hPLCL_A2P_ membranes could accelerate tissue integration and increase ECM protein production, resulting in more rapid and pronounced thickening of pelvic floor fascia. We detected significantly increased hVF proliferation on PLCL_A2P_ and hPLCL_A2P_ membranes, which is consistent with previous studies using supplemented A2P in fibroblast cultures [24, 25, 55]. Moreover, increased metabolic activity of dermal fibroblasts was reported on A2P-releasing PLA scaffolds [24]. We found that the A2P enhanced especially hASC proliferation, being over four times higher on hPLCL_A2P_ than on PLCL, hPLCL and PS control on Day 14. This finding is consistent with previous reports of A2P-induced hASC proliferation [12, 49]. The increased number of fibroblasts, the primary cell type responsible for producing ECM proteins, in addition to other stromal cells, could potentially indicate increased ECM protein production *in vivo*. This, in turn, might be anticipated to potentially enhance the tissue strength and thickness for promoting the support of the pelvic floor fascia. Further, mesh or perforated design is essential for efficient tissue integration *in vivo.* Therefore, we included the perforated hPLCL and hPLCL_A2P_ membranes to represent the possible design for *in vivo* applications. Interestingly, the perforated design of hPLCL_A2P_ appears to further promote hASC proliferation. Similarly, hASC proliferation was also higher on perforated hPLCL compared to PLCL. Such effect of the design was not detected for hVFs. These findings suggest that the perforated design may be beneficial for cell responses *in vitro* as well.

The main ECM components of the pelvic floor tissues are COL I, COL III, and elastin [56, 57]. Supplemented AA and its derivates have shown to increase collagen production of stromal cells [24, 48, 55, 58]. Furthermore, collagen production of dermal fibroblasts was enhanced on A2P-embedded PLA scaffolds [24]. The increased amount of produced ECM proteins, especially COL I, may be anticipated to increase tissue strength, a hypothesis requiring further *in vivo* research. We observed increased hVF collagen production on PLCL_A2P_ and hPLCL_A2P_ membranes using multiple, complementary methods. Extracellular COL I yield was detected in hVFs on PLCL_A2P_ and hPLCL_A2P_ membranes but not on other materials. In addition, both Sircol collagen assay and qRT-PCR show increased hVF collagen production on the A2P-releasing PLCL_A2P_ and hPLCL_A2P_. Interestingly, the amount of total collagen and COL I mRNA was similar between hPLCL_A2P_ and PS control; however, no extracellular COL I was observed on PS. These results are supported by previous findings, where the presence of extracellular collagen in dermal fibroblasts could only be detected the presence of AA [35]. Furthermore, intracellular AA promoted collagen maturation in gingival fibroblasts without increasing mRNA expression [59]. Therefore, we speculate that A2P in PLCL_A2P_ and hPLCL_A2P_ enhances the hVFs’ COL I maturation process rather than solely promoting mRNA expression. Interestingly, extracellular COL I was not previously detected on 3D porous PLCL_A2P_ scaffolds, which might suggest a further effect from the membrane design [36].

Prior research has demonstrated that supplemented AA or A2P increased hASC collagen production and mRNA expression [49, 52]. Furthermore, in the sole study previously published on the subject, A2P-embedded scaffolds promoted hASC collagen production [12]. Accordingly, in this study, we observed that hASCs on PLCL_A2P_ and hPLCL_A2P_ membranes produced a significantly higher amount of collagen and had higher expressions of COL I and COL III mRNA. However, no extracellular collagen was detected on any material. Our findings suggest that A2P enhances collagen production in hASCs but does not have the same effect on COL I maturation, as was seen in hVFs, at least not at the observed hASC maturation state at the 2-week time point. While we detected increased collagen production in both hVFs and hASCs, the overproduction of collagen, especially COL III, may pose the risk of fibrotic growth, which requires further investigation in future experiments. Even though increased collagen production and some degree of fibrosis may provide beneficial mechanical support for the tissue, the excessive formation of fibrotic tissue may lead to scarring, stiffness, and decreased elasticity of the fascia.

POP patients exhibit weakened connective tissue and its altered composition in the pelvic floor, including decreased total collagen content and altered ratios of collagen subtypes [60, 61]. Previous studies have observed elevated levels of COL III and decreased ratio of COL I/III for fibroblasts isolated from POP patients [27, 62]. Moreover, lower proliferation capacity *in vitro* was observed in POP patient hVFs with lower COL I/III ratio (<1.0) [27]. Yet, the published literature on COLI/III ratio is limited and therefore the optimal ratio is currently unknown. In this study, we assessed the ratio of COL I/III mRNA on different materials to investigate the effect of A2P. We detected hVF and hASC COL I/III mRNA ratios <1.0 on all biomaterials tested, except on PS for hVFs and hPLCL for hASCs where the ratio was ∼1.0. However, because the hVFs and hASCs studied here are passaged and cultured *in vitro*, the ratio is only suggestive and rather an observation, and not directly comparative to clinical findings.

Elastin, a major component of pelvic floor connective tissues along with collagen, is essential for normal pelvic floor function. The effect of AA or its derivatives on fibroblast or hASC elastin production is not yet established. Previously, fibroblast elastogenesis was decreased by AA [58] but was enhanced by AA derivative [63]. In our previous research, A2P did not significantly increase hASC elastin expression [12], and according to our knowledge, there is no other previous publications on the effect of A2P on hASC elastin expression. In the current study, we detected increased elastin expression on hPLCL_A2P_ in both hVFs and hASCs. Elastin fibres could provide more elasticity for the forming tissue and therefore possibly preventing the formation of stiff fibrotic scar tissue.

Increased fibroblasts’ expression of α-SMA is considered a marker for myofibroblasts. The amount of myofibroblasts increases during tissue healing as they are the major cell type to produce ECM during tissue regeneration. Therefore, an increased presence of myofibroblasts could provide enhanced support for the pelvic floor [64–66]. In the present study, more α-SMA appears to be present in hVFs on PLCL_A2P_ and hPLCL_A2P_ membranes compared to PLCL and hPLCL membranes, yet the amount of mRNA was significantly higher only compared to hPLCL. The hVFs used in our study were isolated from native vaginal tissue, which are naturally characteristic with α-SMA expressing myofibroblast phenotype. Therefore, they may not be as strongly affected by the A2P in PLCL_A2P_ and hPLCL_A2P_ membranes. However, in a previous study, supplemented AA alone did not increase α-SMA expression in dermal fibroblast [35].

Likewise, α-SMA can be considered as a myofibroblastic marker in hASCs. As a potential therapeutic cell type for POP treatment, we investigated α-SMA expression in hASCs on PLCL_A2P_ and hPLCL_A2P_. We detected significantly higher α-SMA expression in hASCs on PLCL_A2P_ and hPLCL_A2P_. This is in line with our previous study, where A2P-embedded porous PLCL scaffolds increased α-SMA expression in hASCs [12]. Otherwise, previous published research on the effect of A2P on hASC α-SMA expression is limited. Whereas the hVFs may readily be of myofibroblast phenotype, the hASCs still possess great differentiation potential and are not terminally differentiated into specialized collagen-producing fibroblastic cells. Therefore, the A2P may steer the hASCs for acquiring myofibroblastic phenotype to enhance the ECM protein production and tissue healing.

## Conclusions

The novel A2P-releasing PLCL_A2P_ membranes demonstrated promising properties for POP tissue engineering. The A2P-releasing PLCL_A2P_ and hPLCL_A2P_ membranes increased hVF and hASC proliferation and collagen production. However, while the COL I maturation was enhanced in hVFs, hASCs had higher increase in the total production of collagen. Our results indicate hASCs, along with hVFs, to be potential cell types for POP applications. Still, future experiments are required to assess the PLCL_A2P_ performance *in vivo* and to explore the overall necessity of cell-seeding. With these promising *in vitro* results, we aim to further investigate the optimal amount of embedded A2P and the effects of hPLCL_A2P_ on tissue strength and composition *in vivo*.
